# Iron Dysregulation in Mitochondrial Dysfunction and Alzheimer’s Disease

**DOI:** 10.3390/antiox11040692

**Published:** 2022-03-31

**Authors:** John O. Onukwufor, Robert T. Dirksen, Andrew P. Wojtovich

**Affiliations:** 1 Department of Pharmacology and Physiology, University of Rochester Medical Center, Rochester, NY 14642, USA; robert_dirksen@urmc.rochester.edu (R.T.D.); andrew_wojtovich@urmc.rochester.edu (A.P.W.); 2 Department of Anesthesiology and Perioperative Medicine, University of Rochester Medical Center, Rochester, NY 14642, USA

**Keywords:** iron dysregulation, ferroptosis, Alzheimer’s disease, neurodegeneration, mitochondrial dysfunction, reactive oxygen species, lipid peroxidation

## Abstract

Alzheimer’s disease (AD) is a devastating progressive neurodegenerative disease characterized by neuronal dysfunction, and decreased memory and cognitive function. Iron is critical for neuronal activity, neurotransmitter biosynthesis, and energy homeostasis. Iron accumulation occurs in AD and results in neuronal dysfunction through activation of multifactorial mechanisms. Mitochondria generate energy and iron is a key co-factor required for: (1) ATP production by the electron transport chain, (2) heme protein biosynthesis and (3) iron-sulfur cluster formation. Disruptions in iron homeostasis result in mitochondrial dysfunction and energetic failure. Ferroptosis, a non-apoptotic iron-dependent form of cell death mediated by uncontrolled accumulation of reactive oxygen species and lipid peroxidation, is associated with AD and other neurodegenerative diseases. AD pathogenesis is complex with multiple diverse interacting players including Aβ-plaque formation, phosphorylated tau, and redox stress. Unfortunately, clinical trials in AD based on targeting these canonical hallmarks have been largely unsuccessful. Here, we review evidence linking iron dysregulation to AD and the potential for targeting ferroptosis as a therapeutic intervention for AD.

## 1. Introduction

**Figure 1:**
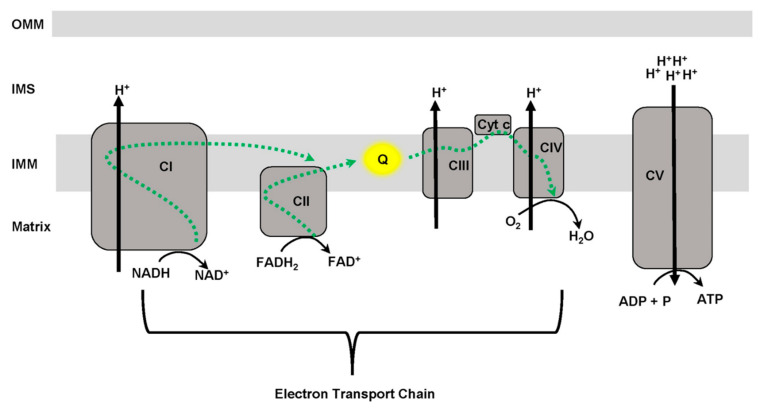
Mitochondrial oxidative phosphorylation (OXPHOS). A mitochondrion is comprised of an outer mitochondrial membrane (OMM), intermembrane space (IMS), inner mitochondrial membrane (IMM) and matrix. Electrons from NADH and FADH_2_ enter the electron transport chain (ETC) at complex I (CI) and complex II (CII), respectively, and are passed to ubiquinone (Q), which then transfers electrons to complex III (CIII). Cytochrome c (Cyt C) transfers electrons from CIII to complex IV (CIV) where O_2_ as the final electron acceptor is reduced to H_2_O. The movement of electrons along the electron transport chain (ETC) results in protons (H^+^) being pump out from the matrix to the IMS at CI, CIII, and CIV. Ultimately, protons are channeled back into the matrix through complex V (CV) where ADP and P are coupled to form ATP.

Alzheimer’s disease (AD) is a progressive age-dependent neurodegenerative disease for which there is currently no cure [1,2]. AD is characterized by continuous deterioration in neuronal activity resulting in impaired cognitive function [1,2,3,4]. This loss of neuronal activity is thought to be multifactorial and result from steady increase in damaged neurons, loss of synapses or neurons, and disruption in neuronal energy homeostasis [1,2,3,4,5,6,7]. Mitochondria supply the bulk of neuronal energy in the form of ATP, primarily through the process of oxidative phosphorylation (OXPHOS) (Figure 1) [8,9,10,11].

Iron is important for neuronal activity and it plays a critical role in myelination, neurotransmitter biosynthesis and energy metabolism [12,13]. Because of its critical importance for neuronal function, iron uptake, distribution, and efflux is highly regulated [14,15]. Cellular iron homeostasis is largely accomplished at the transcriptional level by iron response elements (IRE) and iron regulatory proteins (IRP) [14,15]. Together, both IRE and IRP control the expression of proteins responsible for iron uptake, storage and efflux needed to meet cellular needs [14,16,17,18,19]. Iron dysregulation occurs when there is a disruption in these regulatory proteins and processes. Iron dysregulation can block mitochondrial electron transport chain (ETC) activity and generate reactive oxygen species (ROS) that increase oxidative stress. Iron deficiency or accumulation can lead to cellular dysfunction and energetic crisis.

Damaged mitochondria can increase production of ROS. ROS, when produced in large quantities, can result in oxidative stress and produce oxidative damage to proteins, DNA, and lipids [20,21,22]. Iron dysregulation can also lead to ferroptosis, an iron-dependent form of programmed cell death distinct from other forms of cell death such as apoptosis, necrosis, autophagy, pyroptosis, necroptosis, and parthonatos [23,24,25,26,27,28,29]. Ferroptosis-mediated cell death is caused by uncontrolled ROS-induced lipid peroxidation and can be prevented by glutathione peroxidase 4 (GPx4), an enzyme that converts lipid peroxide to lipid alcohol [28]. Ferroptosis and AD share many features such as the overproduction of ROS, lipid peroxidation, iron overload and reduced energy production. However, AD research has primarily focused on the canonical hallmarks of the disease including amyloid beta (Aβ) and tau. Here we focus on the role of mitochondrial iron dysregulation, ferroptosis, and the potential for targeting neuronal ferroptosis as a therapeutic target for AD.

## 2. Iron Chemistry and Oxidation States

Iron can exist in eight oxidation states from −2 to +6. However, the +2 and +3 states are the most relevant for biological systems [30,31]. In biological systems, iron interchanges between Fe^2+^ and Fe^3+^, which enables iron to act in electron transfer reactions [30,31]. The ability of iron to interchange between oxidized and reduced is an important characteristic that enables iron to participate in various biological reactions. This redox reactivity also confers iron the ability to generate free radicals and highly reactive oxidizing species that are harmful to biomolecules (e.g., proteins, DNA, lipids) [30]. Iron promotes the formation of free radicals through the Fenton reaction whereby Fe^2+^ reacts with H_2_O_2_ to generate a highly reactive hydroxyl radical. Hydroxyl radicals can also be generated from the reaction of superoxide (O_2_^•−^) with H_2_O_2_ through the Haber-Weiss reaction. Haber-Weiss reaction is made possible in the presence of transition metal such as iron [32,33].

Fe^2+^ + H_2_O_2_ → Fe^3+^ + H_2_O + HO^•^ (Fenton reaction)

O_2_^•−^ + H_2_O_2_ → O_2_ + HO^•^ + HO^−^ (Haber-Weiss reaction)

Free iron has the potential to generate large quantities of ROS if left unchecked. Cells protect against this by keeping iron bound to proteins, thereby preventing ROS overproduction. The ability of iron to contribute to redox signaling makes iron important for many metabolic processes, but can also become a destructive force if not tightly regulated.

## 3. Mitochondrial Function

Mitochondria are organelles that generate ATP, the cellular energy currency, through the process of oxidative phosphorylation (Figure 1) [8,9,10,11]. Despite their central role in metabolism, mitochondria also participate in other cellular processes such as Ca^2+^ homeostasis, redox signaling, and cell death, in addition to impacting multiple iron-related roles including iron-sulfur clusters biogenesis and heme syntheses [34,35,36,37,38,39]. Heme, consisting of Fe^2+^ and porphyrin [37,40], is a co-factor of several proteins including cytochrome c, cytochrome P450, hemoglobin, myoglobin, catalases and peroxidases [37,40,41,42]. The role of iron in heme depends on the co-factor and protein. For example, in hemoglobin and myoglobin, Fe^2+^ ensures binding and release of O_2_ during transport. In addition, in electron transfer reactions involving cytochrome c, iron transitions between Fe^2+^ and Fe^3+^. Furthermore, iron in heme containing proteins interchanges between Fe^3+^ to Fe^4+^ during antioxidant activities of catalase and peroxidase. Iron containing proteins participate in numerous cellular activities such as antioxidant defense, oxygen transport and storage, ligand binding, signal transduction, steroid metabolism, gene expression, and redox reactions [40,41,42,43,44,45,46]. Iron-sulfur clusters are protein co-factors consisting of iron and sulfur [47,48,49], possess versatile structural configurations (e.g., 2Fe-2S; 3Fe-4S; 4Fe-4S), and different iron oxidation states (Fe^2+^ or Fe^3+^). The ability of iron to interchange between different oxidation states enables iron-sulfur clusters to participate in a number of biological processes. For example, iron-sulfur clusters play a role in aconitase and succinate dehydrogenase function, citrate acid cycle, proteins involved in DNA replication and repair, redox reaction, nucleic acid processing enzymes, and the transfer of electrons within the ETC [17,47,48,49,50,51].

The mitochondrial ETC is comprised of multiple interacting enzyme complexes (e.g., CI, CII, CIII, and CIV) (Figure 1). The ETC coordinates redox reaction processes whereby electrons from NADH/FADH_2_ are ultimately transferred to molecular oxygen [52]. Throughout this process, protons are pumped from the mitochondrial matrix to the intermembrane space at CI, CIII and CIV [53]. The resulting proton gradient is utilized to drive phosphorylation of ADP to ATP at CV [54,55]. The transfer of electrons down the ETC is coordinated via iron and redox potentials. For example, electrons from NADH at CI are passed from the flavin mononucleotide (FMN) subunit to ubiquinone through eight iron-sulfur clusters [56]. Likewise, CII contains three iron-sulfur clusters that coordinate the transfer of electrons to ubiquinone [57,58]. Lastly, ubiquinone in CIII transfers electrons at the Q_o_ site through an iron-sulfur cluster to cytochrome c [56,59]. In the TCA cycle, the conversion of citrate to isocitrate requires aconitase activation by iron [60,61]. Inactive aconitase contains a 3Fe-4S cluster that upon the addition of iron is converted to the active form containing a 4Fe-4S cluster [60,61,62].

## 4. Mitochondrial Reactive Oxygen Species (ROS)

**Figure 2:**
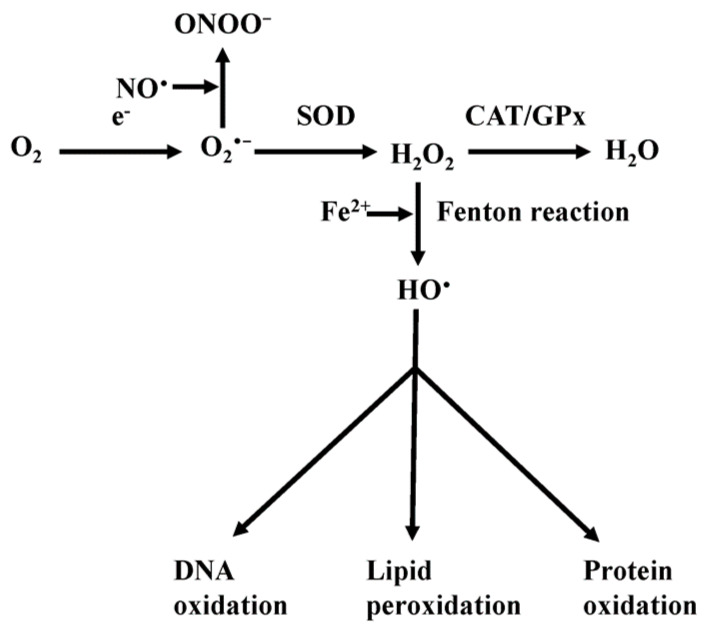
Reactive oxygen species production and toxicity. Oxygen accepts an electron to form superoxide (O_2_^•−^). O_2_^•−^ can react with NO^•^ to form peroxynitrite (ONOO^−^) or be converted to H_2_O_2_ by superoxide dismutase (SOD). H_2_O_2_ can serve as signaling molecule and be converted to H_2_O by glutathione peroxidase (GPx) and catalase (CAT). H_2_O_2_ can also react with transition metals, such as Fe^2+^, through the Fenton reaction to form hydroxyl radical (HO^•^), which if not adequately detoxified, can result in oxidative damage to proteins, DNA and lipids.

ROS are oxygen radical (e.g., O_2_^•−^, HO^•^) and non-radical (e.g., NOX, H_2_O_2_) molecules generated through various cellular processes. Mitochondria are a main site of ROS production as ROS are a byproduct of the oxidative phosphorylation process [20,22,63,64] and ROS levels increase in response to cellular stress conditions such as increased temperature, hypoxia, and heavy metal toxicity [65,66]. Iron alters mitochondrial function through a mechanism that involves impairment of ETC complexes [37,67,68]. Damaged ETC complexes result in an increased number of electrons prematurely reducing oxygen to generate superoxide anion (O_2_^•−^) (Figure 2). O_2_^•−^ is the one electron reduction product of oxygen that is the most common form of ROS produced by mitochondria [21,22]. O_2_^•−^ can either react with nitric oxide (NO^•^) to produce peroxynitrite (ONOO^−^) or be dismutated to H_2_O_2_ by superoxide dismutase (SOD) (Figure 2) [20,21,22,69]. H_2_O_2_ being membrane permeable, thus, can act both locally in the matrix and more distally in the cell following diffusion across the mitochondrial inner membrane [20]. H_2_O_2_ can be detoxified and converted to water by antioxidants and enzyme systems such as glutathione peroxidase and catalase (Figure 2) [20,21,22]. When produced in small amounts, H_2_O_2_ acts as a signaling molecule. However, when produced in large amounts sufficient to overwhelm antioxidant detoxification capacity, H_2_O_2_ can then react with Fe^2+^ through the Fenton reaction to generate highly-reactive HO^•^ (Figure 2) [21,22], which subsequently damages proteins (oxidation), DNA (abstractions/additions), and lipids (lipid peroxidation) (Figure 2).

ROS generated within the proximity of membrane lipids can cause oxidative damage through the initiation of peroxidation of polyunsaturated fatty acids (PUFA), resulting in lipid hydroperoxide. Lipid hydroperoxide can be converted to a non-harmful alcohol product by GPx4 [28]. However, if GPx4 is unable to convert the lipid hydroperoxide to alcohol, lipid hydroperoxide will then undergo a non-enzymatic breakdown to form lipid derived products including 4-hydroxynonenal (4-HNE) or malondialdehyde (MDA) [70,71,72]. ROS can also oxidized proteins forming protein carbonyls [70,73]. Oxidized proteins and lipids can modify multiple diverse cellular function through alterations of protein conformation, inhibition of synthesis of proteins, DNA and RNA [70,71,72]. Indeed, 4-HNE, MDA and protein carbonyls are used as biomarkers of oxidative stress in AD [70,71,72,73,74].

## 5. Mitochondrial Iron Metabolism and Homeostasis

### 5.1. Iron Homeostasis

**Figure 3:**
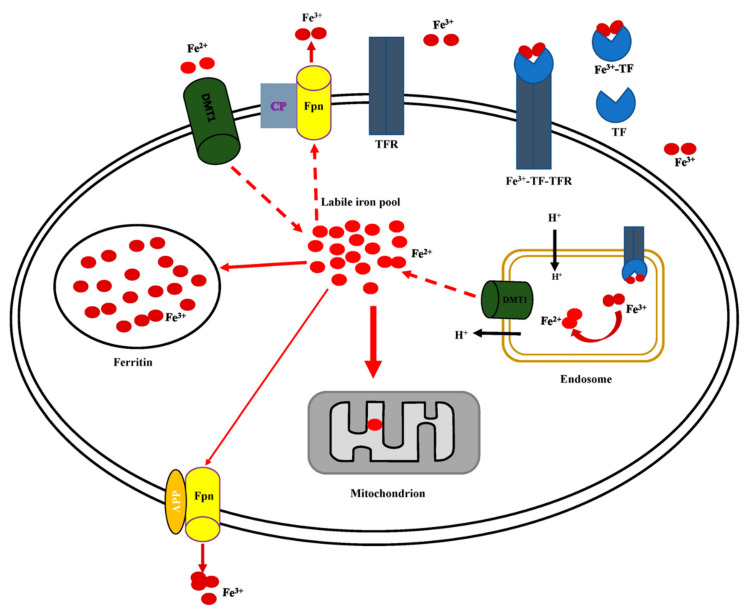
Cellular iron uptake, storage, utilization, and efflux. Extracellular Fe^2+^ is converted to Fe^3+^ through the activity of ceruloplasmin (CP). Two atoms of Fe^3+^ binds to circulating transferrin (TF) and form a Fe^3+^-TF complex. The Fe^3+^-TF complex binds to the transferrin receptor (TFR) and the ternary Fe^3+^-TF-TFR complex is transported inside the cell to the endosome where Fe^3+^ is released. The release of Fe^3+^ is facilitated by the influx of protons (H^+^) that further acidify the endosome. The divalent metal transporter 1 (DMT1), with the aid of H^+^, facilitate the conversion of Fe^3+^ to Fe^2+^. DMT1 then catalyzes the coupled transport of Fe^2+^ and a proton from the endosome to the cytosol. DMT1 can also transport Fe^2+^ directly to the cytosol from the extracellular compartment. Unbound Fe^2+^ in the cytosol forms a labile iron pool that can either be transported to the mitochondrion or stored by ferritin. Excess Fe^2+^ is exported out of the cell through ferroportin (Fpn) with amyloid precursor protein (APP) promoting the conversion of Fe^2+^ to Fe^3+^ before it is released to the extracellular compartment.

In humans, iron content is stable with an estimated 1–2 mg being ingested and a similar amount eliminated daily [12,14,16]. Therefore, there is no net gain or loss of iron under normal homeostatic conditions. This balance is maintained at the cellular level by several regulatory proteins and genes that control iron uptake, storage, utilization, and efflux (Figure 3).

### 5.2. Cellular Iron Uptake, Storage, Utilization, and Efflux

Cellular iron uptake starts with the binding of two Fe^3+^ atoms to circulating transferrin (TF) to form a Fe^3+^-TF complex (Figure 3). When the Fe^3+^-TF complex binds to the transferrin receptor (TFR), the ternary Fe^3+^-TF-TFR complex is transported into the cell, and subsequently, to the endosome [12,14,16,75]. The release of Fe^3+^ in the endosome is coupled with the influx of a proton to reduce the endosomal pH [14,16]. Protons are also required for steap3 and divalent metal transporter 1 (DMT1) oxidation of Fe^3+^ to Fe^2+^. Fe^2+^ is then transported into the cytosol by DMT1 coupled to extrusion of proton, restoring the endosome back to its original neutral condition. While in the cytosol, Fe^2+^ is transferred to mitochondria for use in heme biosynthesis, formation of iron-sulfur clusters, and as co-factors for mitochondrial enzymes [12,14,16,75,76,77].

In addition, Fe^2+^ is stored in a non-toxic form as Fe^3+^ in ferritin. Ferritin has two isoforms: heavy (H) and light (L). Heavy (H) ferritin possess ferroxidase activity and is involved in rapid iron uptake and reutilization. Light (L) ferritin is thought to play role in the nucleation of iron for long term storage. Iron when not stored as ferritin is exported extracellular through ferroportin [14,16].

The export of Fe^2+^ out of the cell by ferroportin requires ferroxidase activities of hephaestin and ceruloplasmin, a multicopper ferroxidase enzyme that oxidizes Fe^2+^ to Fe^3+^ [78]. Thus, ceruloplasmin ensures the release of Fe^3+^ into the circulating system. However not all tissue have ceruloplasmin. For example, in the brain, amyloid precursor protein (APP), an integral membrane protein that acts as a cell surface receptor [79], is utilized for iron efflux outside of neurons [79,80,81].

### 5.3. Regulation of Cellular Iron

**Figure 4:**
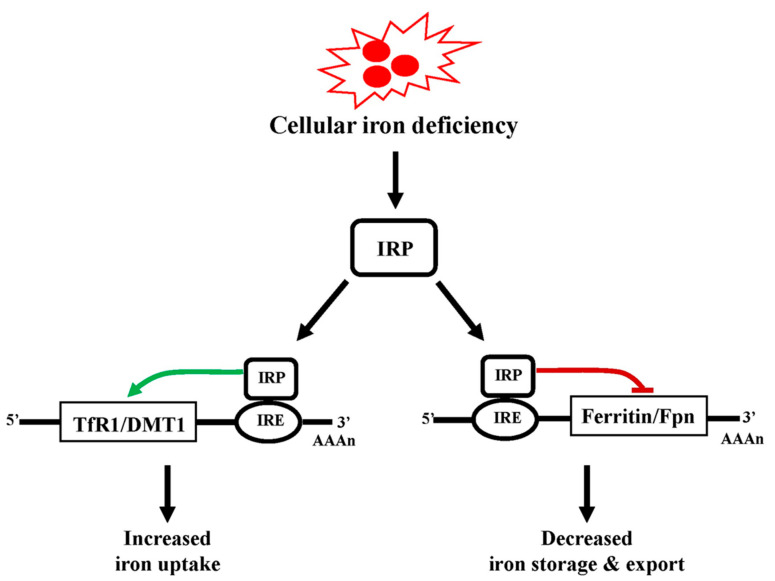
Cellular iron regulation. Deficiency in cellular Fe^2+^ is sensed by iron regulatory proteins (IRPs). Upon sensing decreased iron, IRPs bind to an iron response element (IRE) found in the 3′ untranslated region (UTR) of transferrin receptor 1 (TfR1) and divalent metal transporter 1 (DMT1) mRNA. This binding results in an increased in translation of TfR1/DMT1 proteins leading to increase in cellular Fe^2+^ uptake. IRP also binds to an IRE in the 5′ UTR of ferritin and ferroportin (Fpn) mRNA to block their translation, resulting in reduced iron storage and export.

Cellular iron levels are regulated by the activity of two iron-regulatory protein (IRP) factors that sense the availability of iron [14,15]. Upon iron deficiency, cytosolic IRP factors bind to their respective iron response elements (IRE) to alter the activity of proteins (such as DMT1, TfR1, ferritin and ferroportin) involved in iron uptake, storage and export [14,16,18,19,76]. For example, the binding of IRPs to 3′ untranslated region IREs allows for the upregulation/translation of TfR1 and DMT1 mRNAs to increase iron uptake into the cytosol (Figure 4). On the other hand, IRPs also bind to 5′ untranslated region IREs of the ferritin and ferroportin mRNA to inhibit their translation, thereby reducing production of iron storage and export protein machinery [14,16,18,76]. Given this tight regulation of cytoplasmic iron levels, global changes in iron do not normally occur. However, iron toxicity contributes to disease pathogenesis when localized dysregulation or impairment of these key cellular iron regulatory processes occur.

## 6. Iron and Alzheimer’s Disease

Iron concentrations in the brain increase with age [82,83,84,85,86] and increases in brain iron levels are associated with progressive development of AD [84]. Using the field dependent relaxation rate increase (FDRI) method to quantify iron content of ferritin molecules (ferritin storage of iron in the form of Fe^3+^) and decreased transverse relaxation rate (R^2^) to measure tissue damage, progressive development of AD was shown to be associated with increased brain iron levels [84]. This study found that AD patients exhibited increased Fe^3+^ levels and significant tissue damage in hippocampus compared to the thalamus, consistent with the hippocampus of AD patients being highly susceptible to iron accumulation and damage. Importantly, these results demonstrate that iron accumulation in the brain can be region specific. As a result, some regions of the brain (e.g., hippocampus) are more prone to iron accumulation, and thus damage, than other regions (e.g., thalamus). In line with this observation, brain iron burden correlates well with a decline in cognitive function in AD [87,88,89]. Furthermore, the rate of iron accumulation and its effects on cognitive function are not the same across the different brain regions [85]. Langkammer et al. (2012) used inductively coupled plasma mass spectrometry (ICP-MS) and quantitative susceptibility mapping (QSM) to measure brain iron levels in post mortem AD patients [85]. QSM is a novel magnetic resonance image (MRI) technique that quantifies bulk magnetic susceptibility of a tissue based on tissue generated magnetic fields [82,90,91]. This study found that iron levels varied considerably in different brain regions, with white matter (e.g., frontal) exhibiting lower iron levels (36 mg/kg wet tissue) and gray matter (e.g., globus pallidus) exhibiting the highest levels of iron in the brain (205 mg/kg wet tissue) [85]. Another study used simultaneous quantitative susceptibility mapping and flutemetamol-PET to assess the level of iron and β-amyloid as an indicator of cognitive performance [92]. From 116 older adults with 22% being APOE4 carriers, this study reported a correlation between increased iron and β-amyloid plaques localized in the frontal cortex and temporal cortex [92]. Others also reported an increase in iron levels in both cortex and cerebellum from preclinical AD and mildly cognitive impaired patients [93].

Based on the studies discussed above, cognitive decline in AD patients correlates with age-dependent increases in iron levels in the brain. In addition, different areas of the brain exhibit different degrees of iron accumulation and contributions to cognitive dysfunction in AD. With current advanced imaging techniques, it is now possible to map iron levels within specific regions of the brain and correlate this with cognitive function and AD progression. However, the type of iron (Fe^2+^ and/or Fe^3+^) that contributes to cognitive dysfunction in AD is unknown. To partially address this issue, an in vitro study used a multi-disciplinary approach, including X-ray micro-spectroscopy, X-ray absorption spectroscopy, electron microscopy and spectrophotometric Fe^2+^ quantification techniques, to interrogate the interaction between Aβ1-42 and Fe^3+^ [94]. This study found that Fe^3+^ and Aβ1-42 form aggregates that promote the reduction of Fe^3+^ to Fe^2+^ [94]. Fe^2+^ is a redox active form of iron that reacts with H_2_O_2_ to produced HO^•^, which drives oxidative damage of DNA, proteins, and lipids (see Figure 2).

Several pressing open questions regarding the pathomechanisms of iron toxicity in AD include: (1) How does an increase in iron levels in specific brain regions promote AD progression? (2) What are the underlying molecular mechanisms by which elevated brain iron toxicity correlate with increases in amyloid plaque? (3) Does iron-induced dysfunction in AD result from cellular or intracellular iron dysregulation?

## 7. Mitochondrial Iron Dysregulation and Alzheimer’s Disease

The brain accounts for about 20% of whole body oxygen consumption under resting conditions, despite comprising only about 2% of the mammalian body mass. This indicates that the brain is a highly energetic organ, and thus, is exquisitely sensitive to changes in energy status. Energy dysregulation is among one of the earliest signs of neurodegeneration in AD and is largely attributed to impaired mitochondrial function. Several components of the mitochondrion are altered during neuronal energy crises. Below, we focus on dysfunction in mitochondrial bioenergetics (Section 7.1), mitochondrial fission and fusion (Section 7.2), and the potential impact of iron on mitochondrial function in AD (Section 7.3) [95,96,97].

### 7.1. Dysfunction in Mitochondrial Bioenergetics

Mitochondrial bioenergetics is directly impaired by iron dysregulation through the inhibition of the ETC and indirectly through increased generation of ROS (via mitochondria and NADPH oxidase). Nicotinamide adenine dinucleotide phosphate (NADPH) oxidases are membrane bound proteins that transfer electron from NADPH to oxygen to generate O_2_^•−^ or H_2_O_2_ [98,99,100,101,102]. The generation of ROS by NADPH oxidase are involved in cellular stress response and metabolisms [103,104,105]. Mitochondrial dysfunction in AD is evidenced by decreased mitochondrial membrane potential, elevated ROS levels, increased lipid peroxidation, altered mitochondrial morphology, and increased levels of mitochondrial calcium [95,96,106,107]. AD patients show signs of reduced energy production, an indication that the mitochondrial energetic machinery is compromised [108]. Consistent with this idea, AD patients exhibit significantly reduced expression of nuclear encoded genes for CI, II, III, IV and V subunits compared to that of an aged matched control group [108]. This decrease in ETC protein expression would be expected to contribute to the hypometabolism described in AD. Furthermore, a study that used microarray analyses and quantitative RT-PCR, found that tricarboxylic acid cycle, oxidative phosphorylation, and glycolytic pathways are all significantly downregulated in AD [109]. RNA-seq profiles of post mortem AD brain revealed low expression of insulin receptor substrates, monocarboxylate, acetoacetyl-CoA thiolase, glucose transporters, and pyruvate dehydrogenase as additional contributors to low brain energy observed in AD [110]. Finally, a recent study found that increased activation of NADPH oxidase, an enzyme that uses NADPH to generate superoxide or H_2_O_2_, is a primary mechanism for Aβ1-42 related hypometabolism [111]. Specifically, activation of NADPH oxidase induced hypometabolism in Aβ1-42 models, while inhibition of NADPH oxidase with GSK2795039 abolished hypometabolism [111]. Taken together, these findings indicate that multiple mechanisms contribute to altered energy homeostasis in AD.

### 7.2. Dysregulation in Mitochondrial Fission and Fusion

Mitochondria are dynamic organelles that constantly change their location, length, number, shape and size to meet cellular energy demands [112,113,114]. Mitochondrial dynamics is critical for mitochondrial function and is regulated by a complex network of proteins that control the fusion and fission processes in the outer (OMM) and inner (IMM) mitochondrial membrane [112,113,114,115]. Mitochondrial dynamics ensure maintenance of healthy pools of mitochondria by altering their size and shape to meet energetic demand [112,113,114]. Fusion proteins facilitate the joining of mitochondria to form an elongated mitochondrion. Fusion proteins consists of dynamic GTPase regulator Optic Atropy 1 (OPA1) localized to the intermembrane space, as well as mitofusin 1 (MFN1) and mitofusin 2 (MFN2) proteins located in the outer mitochondrial membrane [112,113,114,115]. Fission proteins promote the splitting or fragmentation of mitochondria. Fission proteins include GTPase regulator Dynamin Related Protein 1 (DRP1), mitochondrial fission 1 protein (Fis1), mitochondrial fission factor (Mff), endophilin-B1, mitochondrial protein 18 (MTP18), and mitochondrial dynamic proteins 49 kDa and 59 kDa [112,113,114,115]. An increase in exogenous Fe^3+^ promotes mitochondrial fragmentation through calcinuerin-mediated dephosphorylation of DRP1 (at ser637) in mouse HT-22 hippocampal neurons [116]. The mechanism is thought to be mediated by iron-induced elevation of intracellular Ca^2+^ and mitochondrial Ca^2+^ overload, which then initiates mitochondrial fragmentation [117,118,119,120]. Similarly, additional studies found that an increase in Fe^3+^ reduced expression of mitochondrial fusion protein OPA1, leading increased mitochondrial fragmentation which resulted in fragmented mitochondria characterized by, mitochondrial number, and total mitochondrial area [97,121]. Indeed, altered DRP1 activity is implicated in increased mitochondrial fragmentation observed in AD [122,123,124,125,126]. Iron-induced energy deficiency in AD is correlated with alterations in the mitochondrial fusion and fission machinery [97,116,121,122,123,124,125,126]. Thus, interventions that target the fission and fusion machinery might represent a viable treatment option for AD.

### 7.3. Linking Iron Induced Mitochondrial Dysfunction in Alzheimer’s Disease

Currently there is a gap in our understanding of how dysregulation in iron alters mitochondrial function in AD. Prior studies have associated iron dysregulation with progression and advance form of AD in both in-vitro and in-vivo [82,83,84,85,86,92,93,94]. Furthermore, several studies (discussed above) found iron dysregulation impacts mitochondrial function and that mitochondrial function altered in AD results in energy crises. However, how iron induced mitochondrial dysfunction leads to AD remains unknown. It will be important for future studies to couple AD models with genetically encoded biosensors that can monitor real-time changes in cellular and intracellular energy status during iron dysregulation in AD. For example, single-wavelength genetically encoded fluorescent sensors for ATP (iATPSnFRs) [127] or NADP^+^/NADPH ratio (iNAP) [128] could be used to quantify bioenergetics changes in AD during iron dysregulation. These sensors enable real-time measurements of cellular ATP levels and redox status in AD model of iron dysregulation, which would provide critical new mechanistic information regarding iron-dependent mitochondrial stress and energy dysfunction in AD.

## 8. Ferroptosis

Ferroptosis is a non-apoptotic mechanism of cell death that is driven by iron dependent lipid peroxidation [23,24,25,26,27,28,29]. Ferroptosis is induced by iron through ROS-mediated lipid peroxidation (Table 1). Similarly, small molecules inhibitors of Xc (such as Erastin, Sorafenib, Sulfasalazine), a cystine/glutamate antiporter, induce ferroptosis (Table 1). In addition, other small molecules that inhibit GPx4 detoxification (such as RSL3, ML162 and NSC144988) also promote ferroptosis (Table 1). Similarly, 4-chlorobenzoic acid, an inhibitor of ferroptosis suppressor protein 1 (iFSP1), induces ferroptosis by blocking activation of CoQ10 (Table 1). In contrast, ferroptosis is inhibited by iron chelators (such as deferoxamine) and activation of CoQ10 (such as idebenone) (Table 1). In addition, ferroptosis is abolished by blocking lipid peroxidation through radical trapping with Ferrostatin1, Liprostatin1, Butylated hydroxytoluene, and α-Tocopherol (Table 1). While many factors can initiate and inhibit ferroptosis, a reduction in GPx4 activity is a hallmark of ferroptosis [28]. GPx4 selectively converts lipid hydroperoxide to a non-reactive lipid alcohol [28]. Induction of ferroptosis requires polyunsaturated fatty acids (PUFA) [129,130]. These PUFA undergo esterification with CoA, with the aid of acyl-CoA synthase 4 (ACSL4), to form phosphatidylethanolamine [129]. Loss of ACSL4 activity prevents the formation of phosphatidylethanolamine, and thus, ferroptosis [129,131,132,133]. Together these findings indicate that ferroptosis requires PUFA esterification. Furthermore, PUFA are also the target of ROS mediated oxidation and subsequent lipid peroxidation. Since ROS-mediated oxidation of esterified PUFA leads to ferroptosis, targeting lipid esterification pathways could be a potential intervention to reduce ferroptosis-induced cell death.

GPx4 activity strongly correlates with ferroptosis. For example, pharmacologic and genetic inhibition of GPx4 activity exacerbates ferroptosis, while GPx4 overexpression ameliorates ferroptosis-induced cell death [134,135,136,137,138]. Recent studies identified ferroptosis suppressors protein 1 (FSP1) as a GPx4-independent mechanism of ferroptosis inhibition [139,140]. FSP1 was formerly known as apoptosis inducing factor mitochondrial 2 (AIFM2) [140,141,142,143]. The mechanisms by which FSP1 inhibits ferroptosis are still under investigation, but is known to involve myristoylation of FSP1 [139,140]. Myristoylation is a protein lipid modification that attaches a 14-carbon unsaturated fatty acid to an N-terminal glycine residue of a subset of proteins [144,145,146]. Upon myristoylation, FSP1 translocates to the plasma membrane where it reduces CoQ10 and acts as a lipophilic radical trap to prevent lipid peroxidation, thus preventing ferroptosis [139,140]. Consistent with this, inhibition of FSP1 exacerbates, and overexpression abolishes, ferroptosis. In addition, FSP1 activity can be modulated pharmacologically by deferoxamine, ferrostatin-1, and idebenone [139,140]. Overall, both GPx4 and FSP1 are two important potential pharmacologic targets to prevent ferroptosis.

**Table 1:** Inducers and inhibitors of ferroptosis.

Small Molecules/Chemicals	Mechanisms/Target	Effects on Ferroptosis	Reference
Erastin	Inhibit system X_c_	Induce	[147]
Sorafenib	Inhibit system X_c_	Induce	[148]
Sulfasalazine	Inhibit system X_c_	Induce	[149]
Glutamate	Inhibit system X_c_	Induce	[150]
RSL3	Block GPx4	Induce	[135]
ML162	Block GPx4	Induce	[136]
ML210	Block GPx4	Induce	[137]
NSC144988	Block GPx4	Induce	[138]
Ferrostatin1	Radical trapping of lipid peroxide	Inhibit	[151]
Liprostatin1	Radical trapping of lipid peroxide	Inhibit	[151]
α-Tocophenol	Radical trapping of lipid peroxide	Inhibit	[23]
Idebenone	CoQ_10_ anolog- Radical trapping of lipid peroxide	Inhibit	[152]
4-Chlorobenzoic acid	Block CoQ_10_ pathway	Induce	[140]
iFSP1	Block CoQ_10_ pathway	Induce	[139]
Deferoxamine	Iron chelator	Inhibit	[23]

## 9. Ferroptosis, Mitochondrial Dysfunction, and Alzheimer’s Disease

**Figure 5:**
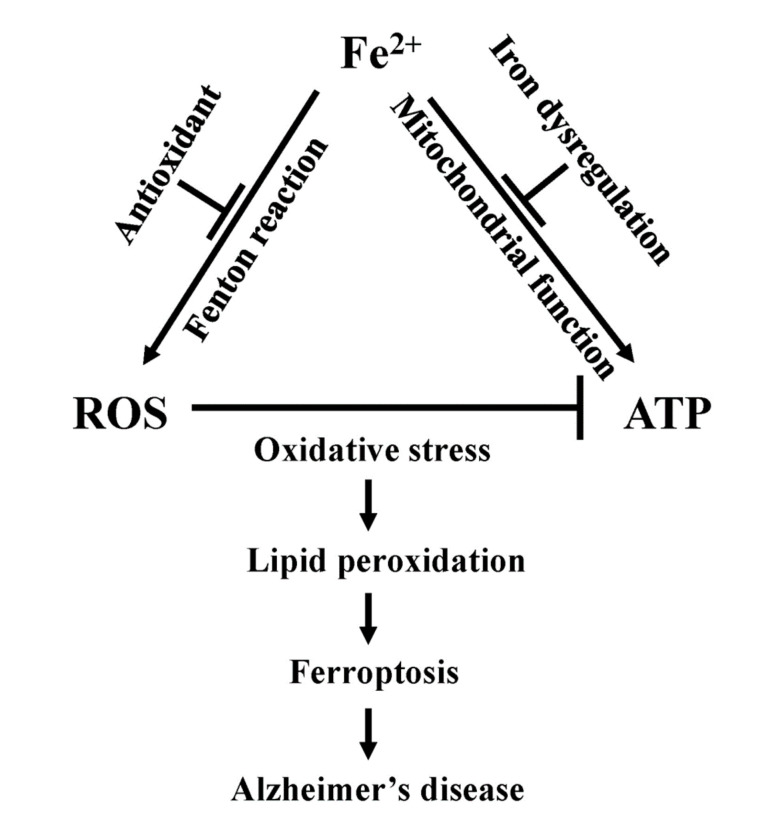
Ferroptosis, mitochondrial dysfunction and Alzheimer’s disease. Unbound Fe^2+^ plays a role in ATP production via electron transfer and enzymatic reactions [12,14,16,75,76,77]. Fe^2+^ dysregulation inhibits mitochondrial function resulting in decreased ATP production [37,67,68]. Fe^2+^ is involved in ROS generation through the Fenton reaction [21,22,32,33] which is blocked by antioxidant [40,41,42,43,44,45,46]. ROS block ATP production via oxidative stress [20,21,22] that damages membrane lipids resulting in lipid peroxidation [70,71,72,73,74]. The accumulation of lipid peroxidation result in cell death known as ferroptosis [23,24,25,26,27,28,29] which can lead to Alzheimer’s disease.

Ferroptosis is linked to many diseases including cancer, ischemia-perfusion injury and neurodegeneration [153,154,155,156,157,158]. However, the precise mechanisms by which ferroptosis is induced in these diseases is unclear. Many of the hallmarks of ferroptosis, such as increased ROS, lipid peroxidation, altered energy status and iron overload are found in AD (Figure 5). For example, an age-dependent downregulation of ferroportin (Fpn) expression is observed in the hippocampus and frontal cortex of APPswe/PS1dE9 AD mice compared to wild-type littermates [159]. In the same study, loss of Fpn occurred concomitant with brain iron overload and atrophy in 9-month-old AD mice [159]. AD is a disease characterized by progressive cognitive decline and memory loss with an inability to perform certain executive functions. Using Morris water maze tests, Fpn KO mice showed reduced learning performance, as well as reduced accuracy, prolong latency in finding the target platform, and a shorter duration in the target quadrant [159]. Furthermore, hippocampal tissue from Fpn KO mice exhibited fragmented mitochondria and higher MDA levels [159]. In addition, GPx4 was downregulated in Fpn KO mice, thus linking a key ferroptosis biomarker to AD progression. In the same study, tissue-specific deletion of Fpn in hippocampus resulted in AD-like hippocampal atrophy and memory deficit [159]. In addition, expression of Aβ in mouse primary hippocampal neurons caused neuronal cell death, while the treatment with ferroptosis inhibitors liprostatin1 or ferrostatin1 prevented cell death. Likewise, over-expression of Fpn in the hippocampus reduced ferroptosis and memory deficits in APPswe/PS1dE9 AD mice [159]. Together, these results provide support for strategies designed to augment Fpn expression and reduce ferroptosis as therapeutic approaches for AD.

Reduced GPx4 activity is a hallmark of ferroptosis and plays critical role in AD pathogenesis [134]. Ablation of GPx4 in mouse hippocampal neurons results in increased levels of lipid peroxidation marker 4-HNE and ERK1/2 phosphorylation, but no change in caspase-3, a marker of apoptosis [134]. These results suggest that the neuronal death observed in GPx4 KO mice was mediated through ferroptosis, not apoptosis. Consistent with this idea, feeding GPx4 KO mice with a vitamin E deficient diet exacerbated ferroptosis, while pharmacological inhibition of ferroptosis with liprostatin-1 protected against neurodegeneration [134]. Neuron-specific knockout of GPx4 results in a rapid onset of paralysis and death that coincides with high levels of 4-HNE, decreased activity of mitochondrial CI and CIV, and motor neuron neurodegeneration, which is delayed when these mice are fed a vitamin E enriched diet [143].

Transgenic overexpression of GPx4 in 5xFAD mice protects 5xFAD mice from developing an AD phenotype [160]. Specifically, 5xFAD/GPx4 mice exhibited reduced lipid peroxidation, improved learning and memory, and higher expression levels of neuronal nuclear proteins. Taken together, these results suggest that overexpression of GPx4 significantly improves neuronal function in the 5xFAD mouse model of AD [160].

Several additional studies employed small molecules as a tool to study neurological damage in AD [161,162,163,164]. In this regard, CMS121, a quinolone derivative that inhibits acetyl-CoA carboxylase1 (ACC1) activity, was shown to prevent AD in APPswe/PS1dE9 transgenic mice [161]. ACC1 is an enzyme that catalyses the conversion of acetyl Coenzyme A to malonyl Coenzyme A, a carbon donor for long-chain fatty acid synthesis [165,166,167]. CMS121 targets and inhibits ACC1, which prevents long-chain fatty acid biosynthesis leading to decreased lipid peroxidation and neuro-inflammation in APPswe/PS1dE9 mice [161]. CMS121 treated APPswe/PS1dE9 AD mice exhibited improved cognitive function as evidenced by reduced escaped latency, reduced time in open arms, and increased time freezing compared to untreated APPswe/PS1dE9 AD mice [161]. In addition, CMS121 treated AD mice also exhibited low levels of 4-HNE and 15LOX2 compared to that of untreated AD mice [161]. As 4-HNE and 15LOX2 are both biomarkers of lipid peroxidation, these findings support the idea that CMS121 reduces lipid peroxidation in AD through inhibition of ACC-dependent production of long-chain fatty acid biosynthesis.

Overall, based on the above studies, mice that lack Fpn and GPx4 exhibit hallmarks of ferroptosis similar to that associated with AD. Moreover, mouse models of AD are characterized by biomarkers of ferroptosis including impaired mitochondrial function and high levels of lipid peroxidation.

## 10. Future Perspectives in Alzheimer’s Disease

AD is a devastating progressive neurodegenerative disease, with patients experiencing signs of impaired memory loss and deterioration in cognitive ability due to progressive neuronal loss [1,2,3,4]. The loss of neural activity reflects the combined effects of sustained oxidative damaged to neurons and reduced energy production in the brain [1,2,3,4,5,6,7]. AD exhibits multiple heterogeneous hallmarks including lipid peroxidation, iron overload, energy imbalance, increased ROS, phosphorylated tau, and Aβ plaques. As a result, a comprehensive understanding of AD pathogenesis requires an integrative approach. However, the majority of prior basic and clinical studies have typically focused on individual phenotypes such as Aβ plaques or phosphorylated tau. The central thesis has been that clearance of Aβ-plaques and phosphorylated tau from the brain of AD patients will be sufficient to restore cognitive function. However, several studies demonstrated that clearance of these oxidized proteins from the brain does not restored cognitive function, while others have shown only slight improvement in cognitive function [168]. These mixed results could in part explain the failure of several clinical trial designs based on the clearance of the plaques from AD patients. For example, the FDA recently approved aducanumab, a monoclonal antibody that targets and binds Aβ plaques to neutralize Aβ toxicity and promote clearance from the brain [1,169,170,171,172]. However, clinical studies indicate that aducanumab does not prevent cognitive decline. Similarly, other drugs purported to reduce Aβ plaques in the brain have also failed to prevent cognitive decline [173,174,175]. While effective in terms of reducing Aβ plaques, treatment with these drugs does not improve cognitive function [168]. Others have proposed reducing Aβ levels via inhibition of beta and gamma-secretase activity. However, this approach has also failed to yield a significant improvement in cognitive function [168]. Given the fact that AD is a heterogenous disease with multiple complex pathogenic mechanisms, it is not entirely unexpected that targeting a single disease hallmark (e.g., only reducing Aβ plaques) has not resulted in significant cognitive improvement. Thus, it is likely that a more integrative approach that addresses multiple pathogenic mechanisms is needed for an effective AD therapy. This integrative approach should be broad enough to mitigate multiple key features of AD pathogenesis including protein aggregation, increased ROS production, reduced antioxidant activity, iron overload, lipid peroxidation and mitochondrial dysfunction.

## 11. Conclusions

In this review, we highlight the need for a paradigm shift in AD research. Specifically, we suggest that the most effect interventions will utilize an integrative approach that combats the complex heterogenous nature of AD. For example, interventions designed to prevent a cellular energy crisis by targeting ferroptosis may provide a novel mechanism to protect against AD. Targeting ferroptosis will unearth previously unknown mechanisms and could serve as a potential therapeutic intervention against AD.
